# Supplementary material for: Are carbon emissions trading and green financial instruments synergistic? -Comprehensive quantitative research based on content analysis
Source: PLoS One. 2024 Mar 7;19(3):e0298601. doi: 10.1371/journal.pone.0298601 (PMC10919601; doi:10.1371/journal.pone.0298601)
Supplement: S1 Data — (ZIP) [file pone.0298601.s001.zip › Data/地级市Z_F工作报告.zip!/╒π╜¡/║■╓▌╩╨/└┤╫╘╣1⁄2╓┌║┼í╛┬φ┐╦╩2╛▌═°í┐_2017.docx]

各位代表：

现在，我代表湖州市人民政府，向大会报告工作，请予审议，并请市政协委员和其他列席人员提出意见。

一、过去五年工作回顾

市七届人大一次会议以来，面对错综复杂的外部发展环境和艰巨繁重的改革发展稳定任务，市政府在省委、省政府和市委的坚强领导下，在市人大、市政协等各方面的监督和支持下，带领政府系统，依靠全市人民，主动适应经济发展新常态，自觉践行新发展理念，创新实干、埋头苦干，谱写了加快建设现代化生态型滨湖大城市的新篇章，湖州大地展现新的面貌，赶超发展开启新的征程。

——这五年，是经济发展奋力赶超的五年。综合实力明显提升，地区生产总值从2011年的1520亿元增加到2016年的2243.1亿元，年均增长8.6%，比全省快0.7个百分点，按常住人口计算，人均达到75715元；三次产业结构比例由7.7∶53.8∶38.5调整为5.7∶47.2∶47.1；财政总收入从219.1亿元增加到360.9亿元，其中地方财政收入从122.1亿元增加到211.2亿元，按可比口径计算，年均分别增长9.7%和10.1%，比全省快1.5和1个百分点。工业强市建设提速提质，规模以上工业主营业务收入由2902.1亿元增加到4217.4亿元，年均增长7.8%；增加值年均增长9.2%，比全省快2.6个百分点，其中战略性新兴产业、高新技术产业、装备制造业年均分别增长12.6%、11.7%和12.5%，比全省快4.3、2.6和4个百分点；长兴、德清相继跨入工业产值超千亿县行列，长兴成为全省工业强县试点和工业转型升级示范区试点；天能、超威、升华、巨人通力、佐力、泰普森、久立、大东吴成为“金象”企业，美欣达、金洲、永兴、德华、欧诗漫、桐昆恒腾成为“金牛”企业。服务业持续快速发展，2016年增加值迈上千亿元台阶，年均增长10%；社会消费品零售总额由609.9亿元增加到1068.9亿元，年均增长12.9%；旅游总收入由263.2亿元增加到882.5亿元，年均增长27.4%，省级旅游度假区实现县区全覆盖，太湖旅游度假区升格为国家级，南浔古镇成功创建国家5A级景区；内河集装箱年吞吐量突破20万标箱，总量位居全省第一，我市获批全国首个内河水运转型发展示范区；德清和安吉入围全省首批服务业强县（区）试点。现代农业和现代林业发展走在前列，在全省农业现代化发展水平综合评价中连续三年位列第一，成为全国第二个基本实现农业现代化的地级市，县区和市级农民合作经济组织联合会相继组建成立，吴兴区在国家现代农业示范区农业改革与建设试点绩效评价中获得第一名。有效投资持续扩大，五年累计完成固定资产投资6278.5亿元，是前五年的两倍，在建百亿级重大产业项目实现县区全覆盖。创新驱动发展战略深入推进，成功创建国家创新型试点城市、国家知识产权示范城市，预计2016年全社会研究与试验发展经费支出相当于地区生产总值的比例达到2.6%，比五年前提高0.78个百分点；“高技术高成长”企业培育行动全面实施，高新技术企业由202家增加到683家；“南太湖精英计划”实施以来累计引进领军型创业创新团队和人才579个，“国千”“省千”人才分别达到38名和99名，累计建成院士专家工作站63家；湖州莫干山高新区升格为国家级，长兴县入列全省首批全面创新改革试验区。

——这五年，是改革开放全面深化的五年。“简政放权、放管结合、优化服务”扎实推进，“四张清单一张网”全面形成，市县区审批同权基本实现，市级部门行政权力事项精减近六成，项目审批提速普遍达到50%以上，全口径财政预算管理体系进一步健全，审计监督全覆盖有序推进，“阳光湖州服务指数”稳步攀升，有力促进了法治政府、服务政府、廉洁政府建设。顺利实施市本级经营性国有资产盘活重组，组建并运行城市、交通、旅游、产业四大国企集团。商事登记制度改革以来，新增市场主体10.9万户，累计达到28.9万户。资源要素配置市场化改革稳步推进，工业企业综合效益分类评价机制初步建立，湖州开发区“四破”试点取得实效，我市获评第三届全国国土资源节约集约模范市。金融和投融资体制改革迈出新步伐，金融机构存贷款余额分别达到3552.4亿元和2757.6亿元，是五年前的1.7倍和1.6倍，银行业信贷资产质量位居全省第二，直接融资规模连续两年超过银行新增贷款、 2016年总量突破700亿元，金融新业态企业达到1310家、管理资本超过1000亿元，新增上市公司10家、累计达到23家。城乡体制改革深入推进，我市入列全国中小城市综合改革试点，德清县和吴兴区织里镇先后入列国家新型城镇化综合试点，省级小城市培育试点实现县区全覆盖，城乡户籍制度改革以及农村“三权”确权、赋权、活权改革率先实现突破，全市城市化率从59.5%提高到62.9%。对外开放水平不断提升，五年累计实到外资超过50亿美元，省外浙商回归到位资金937.4亿元；2016年外贸进出口总额达到679.9亿元，其中出口600.2亿元，年均分别增长4%和4.8%；累计同国外33座城市结成友好关系，企业“走出去”步伐明显加快；接轨沪杭、联通苏皖扎实推进，东西部扶贫协作和对口支援等重大政治任务取得优异成绩。

——这五年，是城乡品质持续提升的五年。基础设施建设力度不断加大，五年累计完成投资1439.2亿元，是前五年的2.4倍。杭宁高铁、杭长高速公路北延、中心城市百里外环线、 318国道改建南浔段等建成通车，商合杭高铁、申嘉湖高速公路西延、杭宁高速公路改扩建、京杭运河航道整治、湖州铁公水综合物流园等全面开建，农村公路改造提升步伐切实加快；太嘉河、环湖河道整治工程基本完工，苕溪清水入湖、扩大杭嘉湖南排工程有序推进；能源、供水、环保、气象、信息化以及教育、卫生、文化、体育等领域一大批项目建成启用或开工建设。湖州中心城市功能品质不断提升，太湖水厂、市民服务中心、湖州儿童公园建成启用，小西街、状元街完成修缮整治，南太湖湿地奥体公园基本建成，湖山大道、浙北医学中心等加快建设，交通拥堵治理持续深化，生活垃圾分类收运处置全面推开。滨湖一体化发展扎实推进，滨湖区域从做基础、做环境向做功能、做产业转变，月亮酒店、龙之梦、影视城等一批项目集聚而来、拔地而起。东部新区、南浔城区、西南分区等发展能级加速跃升。德清、安吉分别入列国家“多规合一”和省级“多规融合”试点，长兴成为全省首批美丽县城试点县。综合行政执法体制改革深入推进，小城镇环境综合治理全面启动，农贸市场标准化改造提升和“放心市场”建设持续深化。美丽乡村建设领跑全省，市级美丽乡村建成率达到80%，省级美丽乡村创建先进县区实现全覆盖，安吉、德清分别以第一和第二名的成绩成为首批省级美丽乡村示范县，由安吉县主导制订的美丽乡村建设指南成为国家标准。“三改一拆”走在全省前列，累计改造旧住宅区、旧厂区、城中村超过1亿平方米，拆除违法建筑3890万平方米。产业平台加速集聚，承载能力切实增强，南太湖产业集聚区等管理体制和运作机制调整优化，全市新形成4个万亩大平台，10个特色小镇进入全省创建和培育名单，吴兴美妆小镇成为省级示范。

——这五年，是生态文明大步前行的五年。“绿水青山就是金山银山”发展理念深入人心，“生态＋”绿色发展方式加快形成，生态环境质量不断提升，立法、标准、体制“三位一体”的生态文明制度体系建设全面推进，在生态省考核中连年获评优秀市，先后成功创建国家森林城市和国家生态县区全覆盖的国家生态市，成为全国地级市首个生态文明先行示范区和生态文明标准化示范区，安吉县荣膺“联合国人居奖”。生态环境治理和保护工作切实加强，五年累计整治关停“低小散”企业4520家，淘汰1182家企业的落后产能，印染、造纸、制革、化工、电镀、铅蓄电池等六大重污染高耗能行业得到大力度的整治和提升，生猪和温室龟鳖养殖业治理工作不断深化，黄标车基本实现清零，节能减排约束性指标全面完成。“五水共治”三夺“大禹鼎”，在全省率先消灭县控以上地表水监测断面劣Ⅴ类和Ⅴ类水质，Ⅲ类以上水质断面比例由88.2%提高到98.7%。 2013年在全省率先开展大气复合污染防治，市区空气质量年优良天数从190天增加到240天，PM2.5年均浓度从每立方米74微克下降到47微克。矿山综合治理不断深化，建筑石料开采量从最高时的1.64亿吨压缩到4800万吨，获批全国绿色矿业发展示范区。耕地资源得到有力保护，连续二十年实现占补平衡。生态文明先行示范区建设条例、市容和环境卫生管理条例相继颁布施行，自然资源资产负债表编制和领导干部自然资源资产离任审计国家试点任务全面落实，自然资源资产保护与利用考核评价等办法制定出台，设立“湖州生态文明日”。美丽经济、绿色产业蓬勃发展，乡村生活、滨湖度假等形成特色品牌，新型动力电池和新能源汽车制造等形成先发优势。

——这五年，是人民生活不断改善的五年。民生投入力度持续加大，全市财政用于民生的支出五年累计达到850.8亿元，占到全部支出的73.9%，我市在第四届中国民生发展论坛上被评为全国地级市民生发展前十强。城乡居民持续增收，城镇居民、农村居民人均可支配收入分别从28993元、 16367元增加到45794元、 26508元，年均增长9.6%和10.1%，家庭人均年收入低于4600元的贫困现象全面消除。就业形势保持稳定，五年累计新增城镇就业34.6万人，2016年底登记失业率为2.52%。社会保障体系更加健全，保障水平逐年提高，被征地农民基本生活保障制度与职工基本养老保险制度实现衔接，新型农村合作医疗制度与城镇居民基本医疗保险制度实现并轨，城乡居民基本医疗保险实现市级统筹，最低生活保障实现城乡同标，职工医疗互助实现扩面提质。住房保障体系建设切实加强，五年累计竣工保障房72745套，完成农房改造7.5万户。城乡社区居家养老服务基本实现全覆盖，每千名老人拥有养老机构床位从21.4张增加到33.5张。基本公共服务均等化水平切实提升，国家义务教育发展基本均衡县区实现全覆盖，成功创建全国首批现代学徒制试点城市和国家特殊教育改革实验区，一类重点大学上线率由12.3%提高到18.2%，教育科学和谐发展业绩水平位居全省第二；我市被列为公立医院改革国家联系试点城市，公共卫生综合发展指数和基本公共卫生服务水平位居全省前列，人均期望寿命从78.3岁提高到80.3岁；“全面两孩”政策顺利实施，出生人口缺陷发生率持续下降；竞技体育和群众体育协调发展，连续举办一系列具有重要影响力的专业赛事，首次成功申办省运会。文化强市建设深入推进，我市成为国家历史文化名城，大运河、“丝之源”、太湖溇港、桑基鱼塘等文化遗产保护和利用工作取得硕果，省级示范文明城市（城区）和文化礼堂建设先进县区实现“满堂红”，公共文化服务体系建设走在前列。社会治理水平不断提升，法治湖州建设全面推进，平安湖州建设实现“十连冠”，南浔、德清、安吉捧得“平安金鼎”，公共法律服务体系基本建立，统一政务咨询投诉举报平台项目获得全国首届法治信访进步奖，安全生产事故起数、死亡人数连续十一年保持下降，圆满完成G20杭州峰会等重大维稳安保任务。

五年来，全市政府系统认真落实全面从严治党要求，坚持讲政治、讲大局、讲激情、讲担当、讲实干、讲自律，切实加强自身建设，统筹推进各项事业。民族、宗教、侨务、港澳、对台等工作取得新成绩，档案、地方志等事业实现新发展，妇女、儿童、老龄、残疾人以及慈善、红十字、志愿服务等事业有了新进步，国防教育、动员、后备力量建设和人民防空、军民融合深度发展等工作得到新加强，实现全国双拥模范城“七连冠”。

各位代表！五年来湖州发展取得的成就来之不易，这是市委总揽全局、正确领导的结果，是市人大、市政协有力监督、全力助推的结果，是全市人民顽强拼搏、砥砺奋进的结果。在此，我代表市人民政府，向奋战在各领域的全市劳动者、建设者和投资者、创业者，向全体人大代表、政协委员、离退休老同志，向各民主党派、工商联、人民团体和社会各界人士，向驻湖人民解放军和武警部队官兵，向所有关心和支持湖州发展的海内外朋友，表示衷心的感谢，致以崇高的敬意！

奋斗曲折艰辛，历程弥足珍贵。实践告诉我们，干大事、创大业，必须敢于担当、勤勉尽责。五年来，全市政府系统紧紧围绕加快湖州赶超发展，强化忠诚、干净、担当，在压力和挑战面前不犹豫、不退缩、不懈怠，坚持改革创新，坚持真抓实干，开辟了各项工作的新境界。在今后的工作中，我们一定要全面强化责任担当，切实拉高发展标杆，不断创造一个又一个实实在在的新业绩。实践告诉我们，干好事、创好业，必须系统施策、精准发力。五年来，全市政府系统紧紧围绕最能够发挥的优势、最需要补齐的短板、最应该突破的瓶颈，把上级部署要求与湖州市情实际紧密结合起来，坚持系统谋划，坚持精准加力，争取了各项工作的主动权。在今后的工作中，我们一定要在把握全局中找准方向，在因地制宜中找对路子，在抓重破难中赢得先机。实践告诉我们，干成事、创成业，必须协同作战、久久为功。五年来，全市政府系统紧紧围绕加快建设现代化生态型滨湖大城市，一个调子喊到底，一张蓝图绘到底，把各方面的智慧和力量凝聚到谋发展、抓建设上来，坚持上下同欲，坚持共建共享，汇成了各项工作的大合唱。在今后的工作中，我们一定要切实增强战略定力，不断增进发展合力，确保各项事业无往而不胜！

各位代表，湖州已经站上赶超发展的历史新起点，但我们也清醒地看到，经济社会发展还面临不少困难，政府工作还存在一些不足。产业结构调整步伐亟待进一步加快，做大经济总量、提升发展质效的任务十分艰巨，引领性强、带动力大的重大产业项目还不多，集聚科技、人才等创新要素的能力还不强，一些企业生产经营仍然比较困难；优质教育、医疗、养老等公共资源供给不充分，空气质量还有不小差距，公共安全领域还有一些短板；转变政府职能、提升行政效能还有很大空间，少数政府工作人员担当精神和执行力还有差距，一些地方和领域消极腐败现象仍时有发生。对这些问题，我们一定高度重视、认真解决，努力把政府工作做得更好，决不辜负人民群众的期望！

二、今后五年工作重点

当前和未来一个时期，世界经济政治正在发生深刻变革，不确定因素明显增加；我国发展处在爬坡过坎的关键阶段，新旧动能正在加速转换；长三角以及全省各地都在卯足干劲、争当标杆，区域竞争更加激烈；我市区位、交通、生态等显性优势和均衡、和谐、文化等内在特质叠加在一起，正在持续释放更加强劲的发展动能，经济社会转型升级、赶超跨越步入十分重要的关口。在百舸争流、竞相发展的大潮中，我们一定要牢牢把握历史方位，咬定目标、抢抓机遇、厚植优势、补齐短板，奋力开创一个动力十足、加快赶超的新局面，建设一个活力迸发、充满希望的新湖州。

按照市第八次党代会作出的部署，今后五年政府工作的总体要求是：高举中国特色社会主义伟大旗帜，以邓小平理论、“三个代表”重要思想和科学发展观为指导，深入贯彻习近平总书记系列重要讲话精神，紧紧围绕“五位一体”总体布局和“四个全面”战略布局，正确把握历史方位，坚定不移贯彻习近平总书记“绿水青山就是金山银山”重要思想和“一定要把南太湖建设好”指示精神，坚定不移以“八八战略”为总纲打好转型升级系列组合拳，坚定不移坚持一张蓝图绘到底，坚持以创新、协调、绿色、开放、共享的发展理念引领赶超发展，主动适应经济发展新常态，以供给侧结构性改革为主线，深入实施“生态立市、工业强市、产业兴市、开放活市”战略，持续开展“四新”主题实践，扎实推进“六重”工作，高质量建设现代化生态型滨湖大城市，高水平全面建成小康社会。

围绕上述要求，重中之重要突出以下五个方面，努力开创发展新局面：

第一，在思想指引上，坚持“三个坚定不移”。坚定不移贯彻习近平总书记“绿水青山就是金山银山”重要思想和“一定要把南太湖建设好”指示精神，就是要高举“两山”旗帜，用好太湖资源，全域融入和全面参与宁杭生态经济发展带建设，以更宽广的视野、更高的站位，全力争取国家和省级重大战略的导入，不断提高湖州元素在全省发展中的影响力和话语权，为把“浙江的今天”建设成“中国的明天”作出更多贡献。坚定不移以“八八战略”为总纲打好转型升级系列组合拳，就是要坚持改革创新不动摇，坚持扬优势、补短板并举，以“拆治归”为基本招数，以创新驱动为核心要领，以有效投资为基础支撑，深入推进符合发展规律、契合区域实际的供给侧结构性改革，加快旧动能的改造提升和新动力的培育壮大，持续释放转型升级的叠加效应。坚定不移坚持一张蓝图绘到底，就是要大力发扬“钉钉子”精神，聚焦落实、落实、再落实，敢于担当、勇于攻坚，注重到位率、平衡性、精细化，困难面前不退缩、问题面前不回避、过错面前不推诿，在确保完成“十三五”规划确定的目标任务基础上，开启基本实现现代化的新征程。

第二，在目标任务上，聚力赶超发展，聚焦“两高”建设。始终把以新发展理念引领赶超发展作为根本任务，努力追求和实现更高质量、更有效率、更可持续的发展速度和发展规模。到届末，地区生产总值突破3000亿元，人均突破10万元，财政总收入突破500亿元，服务业增加值占生产总值的比重超过50%，居民人均可支配收入超过5万元，经济社会发展主要指标人均水平力争达到或超过全省平均。坚持把高质量建设现代化生态型滨湖大城市、高水平全面建成小康社会作为奋斗目标，着力打造创新创业先行区、特色产业集聚区、城乡统筹样板区、生态文明示范区、幸福民生和谐区，彰显“人生只合住湖州”的魅力，实现全面小康“一个不少、一户不落”。经过五年的奋斗，确保政府治理、要素配置等重点领域和关键环节的改革取得决定性突破，大众创业、万众创新的土壤更加肥沃，“4＋3＋N”现代产业体系基本形成，中心城市发展能级和首位度进一步提高，县域经济加快向城市经济转型，美丽城镇建设提档升级，美丽乡村建设继续领先，生态文明先行示范区建设形成更多可复制、可推广的成果，城市文化软实力明显提升，学有所教、劳有所得、病有所医、老有所养、住有所居持续取得新进展，努力让幸福洒落在每一个人身上，让平安洒落到每一处角落。

第三，在发展路径上，深入实施“生态立市、工业强市、产业兴市、开放活市”战略。深化环境整治和生态保护，决不把违法建筑、污泥浊水、“脏乱差”环境带入全面小康，同时加快生态文明制度创新，落实最严格的节能减排约束性机制，实行最严格的环境监管和执法制度，坚持最严格的耕地保护和节约用地制度，培育生态文化，壮大美丽经济，全面提升“生态＋”绿色发展水平，努力率先走向社会主义生态文明新时代。深入贯彻实施《中国制造2025》，培育发展信息技术、高端装备、生物医药等战略性新兴产业，改造提升金属新材、绿色家居、现代纺织、时尚精品等传统优势产业，加快推动“四换三名”和“两化”深度融合，做大做精做强做优实体经济尤其是中高端制造业，努力建设更具实力和竞争力的工业强市。以新技术、新业态、新商业模式改造传统服务业，培育和壮大休闲旅游、健康养生、文化创意、智慧物流、金融新业态等新兴服务业，持续提升现代服务业发展水平。加快培育新型农业经营主体，提高农业科技水平，确保农业现代化发展继续走在前列。推进利用外资、对外贸易、外经合作、服务外包转型升级，深化接轨沪杭、联通苏皖，更好承接产业项目和提供配套服务，更多集聚人才、科技、教育、资本等高端要素，努力提升城市对外开放和国际化水平。

第四，在载体抓手上，持续开展“四新”主题实践，扎实推进“六重”工作，全力主攻高铁枢纽、滨湖一体、全域旅游、新能源整车制造、信息技术产业。以最坚决的姿态、最实干的作风、最担当的精神，纵深推进“四新”主题实践，对齐新标杆，创造新业绩，树立新形象，走好新征程，全力抓好经济、改革、民生、生态、平安、自身建设“六张报表”，努力开创各项事业新局面。以重大项目为牵引、重点工程为龙头、重大平台为载体、重点产业为根基、重大政策为支撑、重点要素为保障，深化和完善“六重”工作年度动态调整、责任分解、考核评估等工作机制，坚持“六位一体”，狠抓落地推进，真正把蓝图化为行动，努力把梦想变成现实。坚持铁、公、水、空协调发展和内畅外联、城乡并举，实施综合交通“一百个项目、八百亿投资”计划，重点打造由杭宁、商合杭、沪苏湖铁路组成的“十”字型高铁枢纽发展格局。以空间布局、规划设计、生态保护、产业协同、基础设施、体制机制“六个一体化”为引领，坚持中心城市和长兴、南浔“一体两翼”组团发展，深入推进滨湖一体融合发展，同时推动德清、安吉组团与滨湖城市带协同发展。以整市打造国家全域旅游示范区为抓手，把健康养生、文化体验、休闲生活深度融入乡村旅游和滨湖度假，持续推进旅游业跨越式发展，加快建设国际生态休闲度假城市。依托动力电池领域的技术先发、产业集群优势和汽车产业其他零部件领域的配套基础，加快引进和培育电机、电控等关键技术和项目，全力进军新能源智能汽车整车制造。继续培育、引进、扶持芯片设计和制造、新型半导体材料研发、地理信息服务等，切实加快产业化步伐，努力抢占新一代信息技术产业发展制高点。同时，突出城市轨道交通、市郊南部山林带规划建设和推动湖州师范学院建设湖州师范大学等，深度谋划和努力办成更多关乎长远的大事和要事。

第五，在导向追求上，践行以人民为中心的发展思想，让全市人民在共建共享发展中有更多获得感。按照人人参与、人人尽力、人人享有的要求，坚守底线、突出重点、完善制度、引导预期，增强政府职责，注重机会公平，保障基本民生，持续提高公共服务共建能力和共享水平，努力使全市人民享有更公平的教育机会、更充分的就业条件、更满意的经济收入、更可靠的社会保障、更舒适的居住环境、更优质的健康服务、更丰富的文化生活、更有力的法治规范、更稳定的公共安全。坚持教育优先发展，优化教育资源布局，深化教育综合改革，全面加快教育现代化建设。实施更加积极的就业政策，鼓励以创业促就业，着力解决结构性就业矛盾。深化收入分配制度改革，加大精准扶贫力度，增强低收入群体发展能力。深入实施全民参保计划，推动社会保障制度更加公平、更可持续。完善住房保障和供应体系，推动住有所居向住有宜居迈进。优化医疗资源配置，深化医药卫生体制改革，促进人口均衡发展，高水平推进健康湖州建设。积极弘扬优秀传统文化，大力培育创新创业文化，完善公共文化服务体系，提升文化产业发展层次。坚持政府带头，强化依法行政，大力推进法治湖州建设，不断提升社会主义法治化水平。坚持“防为上、防为主”，全面提升社会治理现代化水平，努力打造基础更牢、水平更高、人民群众更加满意的平安湖州。凡是广大老百姓牵肠挂肚的、劳心费神的、担惊受怕的、意见集中的公共事务，各级政府和各相关部门必须下更大力气、花更多功夫，查事由、找原因、想办法，努力解决得更好！

三、 2017年工作安排

2017年是党的十九大和省第十四次党代会召开之年，是推进供给侧结构性改革、打好转型升级系列组合拳的深化之年，更是新一届政府履职起步和深化“四个年”、开展“四新”主题实践之年，做好各项工作，意义重大、影响深远。综合考虑各种因素，全市经济社会发展主要预期目标为：地区生产总值增长7.5%以上，固定资产投资增长11%，社会消费品零售总额增长10%以上，外贸出口保持适度增长、提高占全省的份额，财政总收入、地方财政收入均增长7%，城乡居民人均可支配收入分别增长8%和8%以上，节能减排降碳完成省下达任务。工作中，一定要坚持能快则快、应高尽高，全力争取更好结果。

重点做好以下工作：

（一）聚焦产业升级，加快转换发展动能

加快工业经济转型升级。突出绿色制造、智能制造，全力争创“中国制造2025”试点示范城市。以新型电子元器件、地理信息等为重点，促进信息技术产业做大做强，以新能源汽车、物流装备、智能电梯、节能环保装备等为重点，促进高端装备产业做高做新，以生物医药、美妆时尚等为重点，促进现代健康产业做优做深，确保规模以上工业增加值增速快于全省，战略性新兴产业、高新技术产业增加值增速快于面上。以从严对标整治和加快入园集聚、加快改造升级、加快合作转移为路径，继续细分区域、细分行业推动传统产业转型升级。对无证无照、无安全保障、无合法场所、无环保措施的“四无”企业，全面实施专项整治，确保整治和淘汰“低小散”企业（作坊） 1000家以上。实施军民融合产业发展三年行动计划，加快省级军民融合产业基地建设。

提升现代服务业发展水平。推进国家级旅游业改革创新先行区、国家全域旅游示范区、全国内河水运转型发展示范区、全国绿色金融改革创新综合试验区等改革试点，促进旅游、物流、金融等产业创新发展，力争旅游总收入突破1000亿元，内河集装箱吞吐量增长20%以上，绿色金融、现代保险业、金融新业态等培育和发展取得更大突破。大力实施放心消费行动计划，促进消费升级。牢牢把握“房子是用来住的，不是用来炒的”这个定位，继续加强房地产市场科学调控，加快推进非住宅类商品房去库存，着力培育总部型、结算型、创客型楼宇经济，促进房地产业平稳健康发展。大力发展定制经济、工业设计、文化创意和农村电商、跨境电商等，促进现代服务业与先进制造业、现代农业深度融合发展。

推进农业供给侧结构性改革。全面落实粮食安全责任制，积极构建高水平粮食安全保障体系。推进省级农业产业集聚区和特色农业强镇创建工作，推动农业产业结构调整和布局结构优化。加快培育新型农业经营主体，促进农业适度规模经营，培育和认定新型职业农民2000名，新增市级农业龙头企业10家以上、省级和市级示范性家庭农场各50家、示范性农民专业合作社（联合社）20家。推进农业科技创新，深化现代种业工程和农技推广联盟体系建设。大力实施农业机器换人示范工程，加快农业机械化、智能化。深入推进现代生态循环农业建设，深化“打造整洁田园、建设美丽农业”行动，加快畜牧业转型升级，全面淘汰商品龟鳖温室养殖模式，统筹抓好化肥农药减量、废弃农膜回收等工作。实施农业品牌建设三年行动计划，新增无公害、绿色、有机“三品”认证产地面积10万亩。

（二）聚焦实体经济，切实稳固发展根基

扶育实体企业。落实和深化降低要素成本、物流成本、税费成本等各项举措，清理和规范中介服务，扎实有力地帮助企业降本减负。完成小微企业三年成长计划，净增“个转企” 800家、工业“小升规” 135家、服务业“下转上” 100家以上。统筹“金象金牛”大企业和“高技术高成长”企业培育，支持优势企业加大并购重组力度，打造更多行业龙头企业和“隐形冠军”。大力对接多层次资本市场，完成企业股改50家，新增上市公司5家、挂牌企业30家。深入推进“互联网＋”“机器人＋”“标准化＋”，实施企业上云行动、工业机器人行动、质量提升行动，加快质量标准品牌升级，新增上云企业5000家、工业机器人1000台、“浙江制造”标准10个。

扩大有效投资。持续推进“谋划大产业、招引大项目”，围绕全产业链、中心城市功能提升链、培育新动能创新链、全域旅游链抓精准招商，力争实到外资9.5亿美元、省外浙商回归到位资金310亿元，新引进“大好高”项目150个以上，其中制造业项目80个。全面强化项目落地和建设工作，滚动实现“签约引进一批、落地开工一批、建成投产一批”，确保市重点建设实施类项目完成投资735亿元以上。鼓励发展混合所有制经济，积极推进政府与社会资本合作，加强预期引导，增强投资信心，确保民间投资占全部投资的比重稳定在70%左右。扩大重点领域社会投资，确保重大基础设施投资、重大产业项目投资、重点技术改造投资、生态环保投资均增长15%以上。

强化要素保障。拓展融资渠道，优化信贷结构，确保新增融资规模800亿元以上。加强政策性和市场化担保体系建设，推动金融机构加大对制造业、小微企业、“三农”等重点领域的支持力度。严厉打击恶意逃废债和非法集资，加快处置银行不良资产。积极发展股权融资，逐步降低企业杠杆率。运作好政府性产业投资引导基金，有效培育和扶持新产业、新技术、新业态、新商业模式。推进“多规融合”，加快空间拓展，完善基础配套，深化体制创新，不断提高南太湖产业集聚区等各类产业平台集聚化、差异化、特色化发展水平和效益产出。高质量推进特色小镇规划建设。加快实施“3256”耕地保护工程，扎实推进工矿废弃地复垦利用、坡地村镇建设等改革试点，确保新增建设用地指标1.8万亩，盘活存量建设用地6200亩，实施城镇低效用地再开发3500亩，消化批而未供土地7000亩，切实提升节约集约用地水平。

（三）聚焦优势厚植，努力增强发展后劲

扩大生态文明优势。围绕“夺鼎、提标、剿彻底、防反弹”目标要求，深入推进“五水共治”，突出截污纳管、清淤治污、源头防控、防洪排涝等重点治理任务，扎实开展“双千万”行动，全域彻底剿灭劣Ⅴ类水体，监测断面水质100%达到或优于Ⅲ类。深化河长制，健全常态长效管理机制。编制空气质量达标规划，提高雾霾治理科学化、系统化、精准化水平，全面加快用能结构调整，全面淘汰改造燃煤小锅炉，全面完成火电、热电企业清洁化排放提标改造和重点工业企业挥发性有机废气治理，全面加强机动车、船舶污染防治和建筑扬尘防控，全面禁止秸秆露天焚烧，坚决打好蓝天保卫战。从紧从严依法规范管理矿产资源，强化涉矿工程严格监管，加快废弃矿山生态修复，全域推进全国绿色矿业发展示范区建设。坚持预防为主、保护优先、风险管控、分类治理，全面推进土壤污染防治工作。抓好城乡生活垃圾分类收运处置，逐步实现垃圾减量化、无害化、资源化。深入实施“811”美丽湖州建设行动，持续抓好“两路两侧”“四边三化”，推进平原绿化和彩色健康森林建设，加强湿地生态保护和修复。积极探索自然资源产权制度改革，全面实行自然资源资产保护与利用绩效评价、领导干部自然资源资产离任审计和生态环境损害责任追究，加快推进生态文明领域标准化建设，深入实施“生态＋”绿色发展行动计划，不断创新“两山”转化路径，持续扩大生态文明先行示范区影响力。

提高城乡统筹水平。开展新一轮城市总体规划修编工作，推动全域规划向乡镇延伸，完成所有村庄规划修编和中心村村庄设计。大力推进滨湖一体、片区融合、旧城有机更新和智慧城市、海绵城市建设，加快完善教育、卫生、文化、旅游、商贸等功能配套，不断提升中心城市宜居宜业宜游品质。推动三县进一步加大力度，加快打造各具特色的美丽县城。强化“8＋X”项目载体，全面推进小城镇环境综合整治。深化综合行政执法体制改革，下移执法重心，增强管理合力，提高城市和城镇治理水平。加强住宅小区管理，打造美丽家园、幸福社区。推进市校合作共建新农村，加强农村基础设施建设、环境整治和长效管理，再创建一批市级美丽乡村、省级精品特色村和国家级宜居示范村。积极推动东部平原水乡的提升与发展。深化“三改一拆”和“无违建”县区创建，确保完成“三改” 900万平方米、拆违600万平方米。加快城乡体制改革，开展农业供给侧结构性改革集成示范试点，完成农村土地承包经营权确权登记颁证，持续推动农村“三权”活权，促进“三位一体”农民合作经济组织有效发挥作用。

改善基础设施条件。开工建设沪苏湖铁路和杭州绕城高速公路西复线湖州段，加快建设商合杭高铁、申嘉湖高速公路西延、杭宁高速公路湖州段改扩建等工程，全线完成318国道南浔至吴兴段改建工程，推进京杭运河湖州段航道整治、长湖申航道西延等项目，加快农村公路改造提升。开工建设湖长二通道、内环快速路等工程，加快建设湖山大道、南浔三高连接线、五一大桥、苕溪东路拓宽改造等项目，基本完成市区城乡供水一体化建设。基本建成四大重点骨干水利工程，谋划实施苕溪清水入湖河道整治、太嘉河及环湖河道整治、环湖大堤三大后续工程，推进水利工程管理标准化建设。不断提升电力、环保、新能源、信息化等基础设施水平，加快建设长龙山抽水蓄能电站，扎实推动家庭屋顶光伏工程。

（四）聚焦改革创新，持续释放发展活力

深化制度创新。依法平等保护各类产权，大力构建“亲”“清”新型政商关系，让每一位民营企业家都能安心经营、放心发展。深化国企国资改革，推动市属四大国企集团做强做大和县区国有资产盘活重组，推进资产证券化，努力在关键领域发挥关键作用。以企业分类评价结果为依据，加快完善差别化用地、用电、用能、用水等制度体系，切实提高资源要素产出效率。统筹推进吴兴现代农业示范区农业改革与建设试点、南浔城乡发展一体化改革试验区、德清新型城镇化综合试点、长兴全面创新改革试验区、安吉“两山”理论实践示范县等国家级和省级改革试点，全力推动湖州开发区打造产城融合示范区。

推进科技创新。深入实施创新驱动发展战略，争创国家创新型城市、国家可持续发展议程创新示范区。全面建设湖州科技城，加快推进莫干山国家级高新区以及现代物流装备等省级高新区建设，切实提升科技企业孵化器、众创空间等创新平台建设水平。深入实施“双倍增”行动计划，新增高新技术企业70家、科技型中小微企业290家以上。大力推动企业研究院、院士专家工作站等研发机构建设，促进产学研金深度合作，加快新产品、新专利等科技成果产业化。加大“南太湖精英计划”等重点人才工程实施力度，新引进领军型创业创新团队和人才150个左右。深化“9360”急需紧缺人才引进战略合作联盟建设，实施“金蓝领”高技能人才培养工程，出台支持大学生创业政策措施。完善对科技、人才的资金投入、政策保障和绩效评价机制。

加强开放创新。推动外贸品牌、模式、渠道创新，开展外贸“双超”企业培育行动。推进海关特殊监管区建设，提高通关通检便利化水平，加强出口信用保险、国际物流等服务供给，不断优化国际贸易促进体系。深度融入“一带一路”、长江经济带等国家战略，加快湖州铁公水综合物流园建设，整合港口资源，发展河海联运，同时鼓励有条件的企业建设全球营销网络，拓展境外工程市场，深化国际产能合作。深入对接宁杭生态经济发展带规划建设，围绕要素承接、产业发展、制度创新、基础设施共建、公共服务共享等领域，进一步加强与沪、杭、宁等城市的深度合作与融合发展。继续做好东西部扶贫协作和对口支援、友城交流等工作。（五）聚焦民生改善，大力普惠发展成果

加强就业促进和社会保障。坚持以大众创业、万众创新促进充分就业，以加强职业技能培训促进高质量就业，确保城镇新增就业4.8万人以上，登记失业率控制在4%以内。加强和谐劳动关系建设，切实强化企业社会责任，大力培育工匠精神，促进就业稳定有序、工资稳定增长、企业稳定经营。实施集体经济薄弱村消除行动计划，着力补齐低收入农户增收致富短板。加快社会保险参保扩面，推动基本养老保险、基本医疗保险实现法定人群全覆盖，鼓励有条件的企业发展养老保险企业年金。全面实施全市统一的城乡居民基本医疗保险制度和大病保险办法，施行职工基本医疗保险个人账户家庭共济互助政策。加强各类社会救助资源的整合和联动，提高扶贫救困工作精准度。依法推进公益和慈善事业健康发展。实施养老机构规范化管理工程，推进医养结合健康发展。加强残疾人权益保障，加快残疾人共享全面小康进程。

加快社会事业改革与发展。决战全国文明城市创建，深化“最美”系列典型培育，大力弘扬崇德向善的社会风尚。推动教育布局调整优化，扩大优质教育资源覆盖面。加快实施学前教育扩容工程，高水平推进义务教育标准化建设。主动适应高考招生制度改革，不断提升高中段教育质量。推动高等教育、职业教育产教融合，增强服务经济社会发展能力。全面深化公立医院改革，稳步推进医疗机构布局优化。深化医保付费方式改革，深入推进“双下沉、两提升”，加快构建分级诊疗制度。大力发展中医药事业。加强计划生育服务，提高出生人口素质。推进文化场馆资源整合，深化“文化走亲”，积极创建国家公共文化服务体系示范区。加强钱山漾、毗山、龙山古城等遗址保护利用工作。全力筹办筹备第十六届省运会。加快建设浙江自然博物园、浙北医学中心、湖州美术馆、凤西中学、吴兴区人民医院等项目，加快推进双林庆同小学、递铺镇第三小学等一批迁建项目和德清县人民医院、长兴县人民医院等一批改扩建工程，建成湖东小学、滨湖小学、南浔区人民医院等，完成吴兴实验中学、长兴县太湖高级中学等迁建项目。推进医疗、文化、体育等产业多元化发展。

加强和创新社会治理。统筹协调和整体运作社区建设、社会组织建设和社会工作，推进乡镇街道综治工作、市场监管、综合执法、便民服务“四个平台”建设。深化平安细胞创建，畅通群众诉求反映渠道，加强公共法律服务，完善人民调解、行政调解、司法调解对接共融机制，注重矛盾纠纷的源头防范和多元化解。扎实开展“七五”普法，有效增强全社会法治信仰。打造立体化、信息化、法治化社会治安防控体系升级版，严密防范和严厉打击各类犯罪活动。深化户籍制度改革，推行新型居住证制度。深化安全生产体制机制创新，建立重点行业风险防控和隐患治理双重预防体系，确保安全生产形势持续平稳向好。全面实施“除险安居”行动，加快城乡危旧房屋治理改造和地质灾害隐患综合治理，推进防汛防台、防灾减灾体系建设。加快建立食品药品质量追溯制度，切实让老百姓吃得安全、用得放心。加强国防动员、国防教育、基层武装和人民防空建设，推进新一轮全国双拥模范城创建工作，扎实做好民族、宗教、侨务、对台等工作。

各位代表！增进人民福祉，是政府一切工作的出发点和落脚点。我们一定要从解决群众最关心、最直接、最现实的利益问题入手，竭力办好各项民生实事。今年，我们按照“群众提、代表定、政府办、人大评”的理念，在广泛征询意见建议的基础上，梳理形成十二个民生实事候选项目，提请本次大会票决选出其中十个。民生实事项目确定后，我们将紧扣目标要求，认真组织实施，确保高标准、高质量全面完成。

四、全面从严加强政府自身建设

事业关键在人，赶超根本靠干。站在历史新方位，开创发展新局面，迫切需要我们以对党、对人民、对湖州极端负责的精神，以深入推进“两学一做”学习教育常态化制度化、扎实开展“四新”主题实践为统领，全面从严加强政府自身建设，努力打造忠诚履职、依法行政、干净干事的法治政府、服务政府和廉洁政府。

（一）牢固树立“四个意识”，切实做到担当有为。全面增强政治意识、大局意识、核心意识、看齐意识，在思想上政治上行动上坚决同以习近平同志为核心的党中央保持高度一致，不折不扣贯彻省委、省政府和市委各项决策部署，大力提升政府执行力、公信力和创造力。坚持重实干、讲实绩、求实效，建立重点工作清单制度，完善政府绩效考核评价体系。加强政务诚信建设，撬动全社会信用体系建设。建立健全正向激励和容错纠错机制，加大对懒政怠政行为的追责问责力度，严厉整肃行政不作为、慢作为、乱作为和“圆滑作为”“模糊作为”，让敢于担当、善于担当蔚然成风，打造致力创新破难、奋力赶超发展的政府铁军。

（二）不断深化政府改革，大力提升行政效能。进一步加大“放管服”改革力度，大幅减少行政审批事项，大幅缩短审批服务时间，大幅下放行政审批权限，大幅提升网上审批能力，大幅降低审批服务费用。深化“两集中、两到位”和“互联网＋政务服务”，全面推进“最多跑一次”改革，确保今年底前全市“最多跑一次”事项覆盖率达到90%以上，其中“零上门”事项覆盖率40%以上。巩固和扩大“先照后证”改革成果，推动“区域能评、环评＋区块能耗、环境标准”逐步取代项目能评、环评，实现所有投资项目网上审批、核准、备案。全面推行“双随机、一公开”，杜绝多头执法、重复检查，形成“部门联合、随机抽查、按标监管”的“一次到位”机制。加快投融资体制机制创新，加强政府性债务管理。

（三）全面加强依法行政，加快转变政府职能。健全行政权力监督制约机制，自觉接受人大依法监督、政协民主监督和监察监督、司法监督，主动接受社会监督、舆论监督，切实加强政府内部层级监督和审计监督。严格执行重大行政决策程序规定，完善重大事项公众参与、专家论证、合法性审查等制度，充分发挥政府法律顾问、决策咨询等制度作用，加大政务公开力度，推进阳光政府建设。出台市政府规章制定程序规定，加强重点领域政府立法工作。全面落实行政执法责任制，推进行政复议体制改革。高质量完成政府职能转变三年任务，全面开展服务型政府建设行动，统筹抓好向社会组织转移职能、向社会购买服务工作。

（四）严格落实主体责任，深入推进廉政建设。牢牢抓住主体责任这一“牛鼻子”，坚持“一岗双责”、以上率下，层层传导压力，扎实推动全面从严治党向纵深发展，大力营造风清气正的良好政治生态。高标准落实中央八项规定精神，驰而不息正风肃纪，坚决防止“四风”反弹回潮。以公共资金、公共资源、国资国企、机构编制等为重点，全面加强规范管理，扎紧织密制度笼子。坚持政府过“紧日子”、让企业和群众过“好日子”，严格预算管理，优化支出结构，提高财政资金使用绩效。保持惩治腐败高压态势，全力支持纪检监察机关监督执纪问责，坚决查处侵害群众利益的不正之风和腐败问题。

各位代表！赶超的使命令人奋发，人民的重托催人奋进。站在新起点，建设新湖州，我们满怀信心、豪情万丈。让我们在中共湖州市委的领导下，万众一心，开拓进取，“撸起袖子加油干”，为高质量建设现代化生态型滨湖大城市、高水平全面建成小康社会而努力奋斗！
